# Real-world outcomes in patients with brain metastases secondary to HR^+^/HER2^−^ MBC treated with abemaciclib and local intracranial therapy

**DOI:** 10.1093/oncolo/oyae274

**Published:** 2024-10-17

**Authors:** Wambui Gathirua-Mwangi, Holly Martin, Dan He, Shen Zheng, Kristin M Sheffield, Jincy John, Erika Yamazawa, Sarah Rybowski, Priscilla K Brastianos

**Affiliations:** Eli Lilly and Company, Indianapolis, IN, United States; Eli Lilly and Company, Indianapolis, IN, United States; Syneos Health, Morrisville, NC, United States; TechData Service Company LLC, King of Prussia, PA, United States; Eli Lilly and Company, Indianapolis, IN, United States; Eli Lilly and Company, Indianapolis, IN, United States; Harvard Medical School, Massachusetts General Hospital, Boston, MA, United States; Eli Lilly and Company, Indianapolis, IN, United States; Harvard Medical School, Massachusetts General Hospital, Boston, MA, United States

**Keywords:** brain metastasis, abemaciclib, HR^+^/HER2^−^, treatment patterns, breast cancer, real-world outcomes

## Abstract

**Background:**

Real-world data are limited for patients with brain metastases secondary to metastatic breast cancer (MBC) and treated with cyclin-dependent kinase 4/6 inhibitors (CDK4/6i). This study describes real-world outcomes in patients with hormone receptor-positive, human epidermal growth factor 2-negative (HR^+^/HER2^−^) MBC with brain metastases diagnosis before abemaciclib initiation.

**Patients and Methods:**

A nationwide electronic health record-derived de-identified MBC database (January 2011-December 2021) was assessed retrospectively. Patients with HR^+^/HER2^−^ MBC who were treated with abemaciclib (monotherapy or in combination) following diagnosis of brain metastases were included. Real-world best response reflected clinician-documented response assessment of the brain imaging (intracranial) and change in disease burden following radiographic imaging (extracranial); these were reported descriptively. Time to treatment discontinuation (TTD), real-world progression-free survival (rwPFS), and overall survival (rwOS) were assessed using Kaplan-Meier methods from abemaciclib initiation (index date).

**Results:**

Among 82 included patients (mean age 57.0 years; 98.8% female), 22.0% and 19.5% received CDK4/6i and chemotherapy before abemaciclib initiation, respectively, and the majority (80.5%) received radiation/local surgery to the brain before abemaciclib initiation. Patients mostly received abemaciclib as monotherapy (*n* = 6) or in combination with endocrine therapy (*n* = 68). Median TTD was 7.1 (95% CI 4.6-11.3) months, rwPFS was 9.2 (95% CI 6.0-11.6) months, and rwOS was 20.8 (95% CI 13.9-26.0) months. Intracranial and extracranial objective response rates, as determined by treating physicians, were 45.1% (*n* = 23/51) and 56.7% (*n* = 34/60), respectively. Intracranial and extracranial clinical benefit rates were 62.7% (*n* = 32/51) and 70.0% (*n* = 42/60), respectively.

**Conclusion:**

In this real-world study of patients diagnosed with brain metastases and initiating abemaciclib, most patients received radiation/local surgery to the brain before abemaciclib initiation. Although the outcomes in this real-world study are encouraging, it is unclear if the benefit was due to local therapy, abemaciclib, or the combination, and causality cannot be inferred. Further prospective clinical studies are needed to confirm the clinical benefit of this approach.

## Teaser

This article describes patient characteristics, treatment patterns, and real-world outcomes of abemaciclib-treated patients with brain metastases in HR^+^ /HER2^−^ metastatic breast cancer

**Implications for Practice**

In a previous phase II trial (NCT02308020), abemaciclib demonstrated clinical benefit in a subset of patients with HR^+^ metastatic breast cancer (MBC) with brain metastases, with a decrease in intracranial lesion size in 38% of patients and an intracranial clinical benefit rate of 24.1%. However, the trial did not meet its primary endpoint. The current real-world study suggests potential clinical activity of abemaciclib-containing regimens, often in combination with radiation or surgery, with regards to intracranial and extracranial outcomes, time to treatment discontinuation, real-world progression-free survival, and real-world overall survival in patients with brain metastases in HR^+^/HER2^−^ MBC. However, further confirmatory clinical studies are needed.

## Introduction

By 2025, over 169 000 individuals in the United States are projected to be living with metastatic breast cancer (MBC).^1^ Hormone receptor (HR)-positive, human epidermal growth factor 2 (HER2)-negative (HR^+^/HER2^−^) breast cancer is the most common breast cancer subtype in the United States with an age-adjusted incidence rate of 87.2 cases per 100 000 women, per 2016-2020 data.^2^ The incidence of brain metastasis is 15% among patients with HR^+^/HER2^−^ MBC,^3^ and the prognosis for these patients is poor. Analysis of SEER-Medicare data reported worse median overall survival (OS) among patients with HR^+^/HER2^−^ MBC brain metastasis (9.4 months) versus no brain metastasis (26.1 months).^4^ Moreover, the presence of brain metastasis was associated with higher all-cause mortality rate and MBC-related costs.^4^

Brain metastases in HR^+^/HER2^−^ MBC are treated in a multidisciplinary way with surgery, radiotherapy, and/or systemic therapy.^5,6^ Surgery is reserved for patients with solitary brain lesions, lesions associated with significant mass effect, large posterior fossa tumors, or in situations where tissue diagnosis is needed.^7^ Stereotactic radiosurgery (SRS) is recommended for patients with limited or resected brain metastases. For patients with symptomatic brain metastases, local control with surgery and/or radiation is strongly recommended.^8^ Systemic therapy such as chemotherapy, endocrine therapy (ET), and cyclin-dependent kinase 4/6 inhibitors (CDK4/6i) are also commonly used.^6,9^

Abemaciclib is a CDK4/6i approved for use in HR^+^/HER2^−^ MBC as monotherapy or along with an aromatase inhibitor (AI) or fulvestrant, based on the results of the MONARCH trials.^10-12^ Other CDK4/6i such as palbociclib and ribociclib are also approved for use in HR^+^/HER2^−^ MBC in combination with an AI or fulvestrant, based on results from the PALOMA^13,14^ and MONALEESA trials.^15,16^ Abemaciclib has demonstrated brain penetration activity in animal models^17,18^ and in a physiologically based pharmacokinetic model.^19^ Tolaney et al^20^ evaluated the effect of abemaciclib in patients with HR^+^ MBC with brain metastasis in a phase II study. In this study, in a cohort of patients with brain metastasis secondary to HR^+^/HER2^−^ MBC, the intracranial objective response rate (ORR) was low at 5.2% (95% CI 0.0%-10.9%), the intracranial clinical benefit rate (CBR) was 24% (95% CI 13.1%-35.2%), and the median overall survival (mOS) was 12.5 months (95% CI 9.3-16.4). Additionally, 38% of patients experienced a volumetric decrease in target intracranial lesions, although this did not meet the criteria for partial response. Although the study did not meet its primary endpoint of a confirmed intracranial ORR, there was some evidence that suggested abemaciclib had some clinical benefit in a subset of patients, thus leading to interest in further evaluation of abemaciclib among patients with brain metastasis in MBC.

Real-world data are useful to understand patient outcomes in routine clinical practice.^21^ Goyal et al^4^ reported lower mOS in patients with HR^+^/HER2^−^ brain metastasis. Previous studies in patients with brain metastasis in MBC have demonstrated favorable prognosis in those who received radiotherapy, craniotomy, or targeted therapy.^22-26^ Additionally, a single institution study from the United States reported a mOS of 36.7 months in patients with brain metastasis in HR^+^ MBC who received stereotactic radiotherapy and CDK4/6i.^27^ However, real-world data specifically for abemaciclib-treated patients with brain metastasis in HR^+^/HER2^−^ MBC are limited. Therefore, the present study aims to describe the patient characteristics, treatment patterns, and real-world outcomes of abemaciclib-treated patients with brain metastasis in HR^+^/HER2^−^ MBC.

## Materials and Methods

### Data source and study design

This observational, retrospective, descriptive, cohort study used data from the nationwide Flatiron Health electronic health record (EHR)-derived de-identified database from January 1, 2011, through December 31, 2021. The Flatiron Health database is a longitudinal database, comprising de-identified patient-level structured and unstructured data, curated via technology-enabled abstraction.^28,29^ The de-identified data originated from approximately 280 US cancer clinics (~800 sites of care). The database includes structured and unstructured EHR data elements such as patient demographics; clinical characteristics (clinical diagnoses, cancer stage at diagnosis, site of metastases, performance status, and laboratory and biomarker data); treatments received (medications administered, local treatments such as radiation, surgery and oncologist-defined and rule-based lines of therapy [LoT]); and treatment outcomes, clinician-documented treatment response, and month and year of death.

This observational study only used previously collected data, did not impose any form of intervention, and de-identified the data to protect participant privacy. Therefore, a formal Consent to Release Information form was not required. This study was conducted in accordance with the ethical principles that have their origin in the Declaration of Helsinki and that are consistent with Good Pharmacoepidemiology Practices^30^ and applicable laws and regulations of the United States.

Index date was defined as the date of initiating abemaciclib between September 1, 2017, and September 30, 2021 (index period) to allow for ≥90 days of follow-up until December 31, 2021.

### Patient population

The study included patients who had evidence of stage 4 de novo or recurrent MBC, and evidence of HR^+^/HER2^−^ any time pre-index to 30 days post-index; had ≥2 recorded visits in the Flatiron Health database after index; initiated treatment with abemaciclib in the MBC setting on or after September 1, 2017; had brain metastasis diagnosis prior to abemaciclib initiation; were ≥18 years of age at index date; and had ≥90 days of follow-up period from index date through loss of follow-up (ie, confirmed last activity in EMR) or study end date (December 31, 2021; Supplementary Appendix 1). Patients who died within 90 days post the index date were also included.

The study excluded patients who were treated with abemaciclib >14 days prior to MBC diagnosis date; evidence of <7-day supply of abemaciclib; had HER2^+^ disease as evidenced by receiving HER2^+^ targeted therapy at any time pre-index to 30 days post-index; were treated with a clinical study drug in combination with abemaciclib in the first abemaciclib LoT in the MBC setting; or had a diagnosis of primary malignancies other than MBC while being treated for MBC (Supplementary Appendix 1).

### Outcomes and variables

Baseline demographic and clinical characteristics as well as Charlson’s Comorbidity Index (CCI),^31^ Eastern Cooperative Oncology Group (ECOG) Performance Status (PS), menopausal status, and lab and biomarker tests; and MBC-related variables including presence of brain metastasis at index date and time from MBC diagnosis to abemaciclib initiation were assessed from structured data or abstracted from the EHR. Trained medical data abstractors performed data abstraction by reviewing the unstructured real-world data.

Treatment-related variables including the patients’ index abemaciclib-based regimens, follow-up time, LoT details, and regimens received prior to index abemaciclib were evaluated. Time to treatment discontinuation (TTD) was defined as the time from the index date to the abemaciclib discontinuation date which was determined by (1) a patient having a gap of ≥60 days without abemaciclib, (2) initiation of a subsequent LoT, or (3) death, whichever was earliest. Patients who did not discontinue index abemaciclib were censored at the last abemaciclib observed date. Reasons for treatment discontinuation were also evaluated.

Real-world outcomes including the presence of brain lesions and intra- and extracranial outcomes such as ORR and CBR were evaluated. The response was based on the clinician-documented assessment of the brain imaging (intracranial) and the change in disease burden following radiographic imaging (extracranial) within the abemaciclib LoT. Trained medical data abstractors abstracted intracranial response data for all patients, and those with no clinician documentation were noted. Clinicians verified and validated the response. This approach was evaluated in a feasibility analysis prior to initiating the current study. Thirty patients were randomly selected in this feasibility analysis to determine (1) the availability of radiographic imaging of the brain, (2) the completeness of clinician-documented response assessment(s) of the brain imaging, and (3) how clinicians document response in the brain in the real-world setting. Results demonstrated that the majority of patients had evidence of at least one brain imaging-based assessment and clinician-documented response assessment during the abemaciclib line of therapy. Based on the feasibility analysis, policies and procedures were developed to guide the abstraction team to consistently capture these outcomes. The best response was selected in this order: complete response, partial response, stable disease ≥24 weeks, stable disease <24 weeks, radiation necrosis, progressive disease, indeterminate response, and not documented. ORR was defined as the proportion of patients with documented complete response or partial response ≥30 days after the index date and before/on the end date of index abemaciclib. CBR was defined as the proportion of patients with documented complete response, partial response, or stable disease ≥24 weeks after the index date.

Real-world progression-free survival (rwPFS) was defined as the time from the index date to progression, transition to hospice, or death (whichever occurred first). Patients without one of these events were censored at the last date of drug administration in the LoT. Real-world overall survival (rwOS) was defined as the time from the index date until death. Patients without a death event were censored at the last patient visit in the database.

### Statistical analysis

Patient characteristics, treatment patterns, and intra- and extracranial outcomes were evaluated descriptively. All time-to-event analyses were conducted using the Kaplan-Meier method.

Patients were classified into 4 subgroups: abemaciclib + aromatase inhibitors (AI: letrozole, anastrozole, or exemestane); abemaciclib + fulvestrant; abemaciclib monotherapy; and abemaciclib + other (ie, abemaciclib without an AI or fulvestrant upon initiation of abemaciclib therapy). If an AI and fulvestrant were present in the same LoT, the patient was categorized as abemaciclib + other. The assigned subgroup was not changed despite use of luteinizing hormone-releasing hormone drugs such as goserelin or leuprorelin. Patient characteristics and treatment patterns were presented for the overall cohort and by subgroup. Extracranial and intracranial outcomes were presented only for the overall cohort. All analyses were conducted with SAS Software v9.4.

## Results

### Demographics

A total of 82 patients met the inclusion and exclusion criteria (Supplementary Appendix 1). The majority of the cohort were female (*n* = 81), and the mean (SD) age was 57.0 (12.2) years (Table 1). Of all the patients, 56.1% were White and 59.8% lived in the Southern United States and Western regions of the United States. Approximately 89.0% of patients initiated abemaciclib in the community practice setting and 30.5% had commercial-only type of health insurance. The median (interquartile range [IQR]) follow-up time was 12.8 (5.8-21.0) months.

**Table 1.** Demographic characteristics.^a^

Characteristic	Total (*N* = 82)	ABE + AI (*n* = 28)	ABE + FUL (*n* = 40)
Age at index date in years, mean (SD)	57.0 (12.2)	55.4 (12.3)	57.0 (12.6)
Age group at index date in years, *n* (%)			
≤49	21 (25.6)	8 (28.6)	10 (25.0)
50-64	41 (50.0)	15 (53.6)	20 (50.0)
65-84	20 (24.4)	5 (17.9)	10 (25.0)
Sex, *n* (%)			
Female	81 (98.8)	28 (100.0)	40 (100.0)
Male	<5	<5	<5
Race, *n* (%)			
White	46 (56.1)	15 (53.6)	25 (62.5)
Other Race	17 (20.7)	9 (32.1)	6 (15.0)
Black or African American	11 (13.4)	<5	5 (12.5)
Missing	8 (9.8)	<5	<5
US region, *n* (%)			
South	31 (37.8)	11 (39.3)	16 (40.0)
West	18 (22.0)	9 (32.1)	6 (15.0)
Northeast	11 (13.4)	<5	5 (12.5)
Midwest	11 (13.4)	<5	5 (12.5)
Missing	11 (13.4)	<5	8 (20.0)
Practice setting, *n* (%)			
Community	73 (89.0)	25 (89.3)	34 (85.0)
Academic	8 (9.8)	<5	6 (15.0)
Both	≤5	<5	<5
Insurance payer category before or on the index date, *n* (%)			
Commercial only	25 (30.5)	11 (39.3)	9 (22.5)
Public with/without commercial	14 (17.1)	5 (17.9)	5 (12.5)
Other	28 (34.1)	9 (32.1)	17 (42.5)
Missing	15 (18.3)	<5	9 (22.5)
Follow-up time in months from the index date, median (IQR)	12.8 (5.8-21.0)	13.5 (5.5-21.4)	13.9 (6.9-20.9)

Insurance includes public (Medicaid, Medicare, and other government program), commercial (commercial health plan), and other (other payer—type unknown, patient assistance program, and with/without public/commercial).

^a^Fourteen patients received either abemaciclib monotherapy (*n* = 6) or abemaciclib in combination with other (*n* = 8). Due to the small sample size and risk of patient re-identification, the data for these subgroups were not presented.

Abbreviations: ABE, abemaciclib; AI, aromatase inhibitors; FUL, fulvestrant; IQR, interquartile range; *N*, total number of patients; *n*, number of patients in the subgroup; PR, Puerto Rico.

In the overall cohort, patients received abemaciclib in combination with AI (*n* = 28) or fulvestrant (*n* = 40). The demographic characteristics of these subgroups (abemaciclib + AI, abemaciclib + fulvestrant) were generally similar to the overall cohort (Table 1). Due to the small sample size in patients receiving abemaciclib monotherapy (*n* = 6) and abemaciclib + other (*n* = 8), and the risk of patient de-identification, data for these subgroups were not reported.

### Clinical characteristics

Overall, 75.6% of patients had stage 1-3 breast cancer at initial diagnosis, at index 13.4% had an ECOG PS score of 2+, 19.5% had a missing ECOG PS score, 39.0% had CCI ≥ 1, and 61.0% were overweight or obese (Table 2). Patients initiated abemaciclib a median of 5.5 (IQR 1.8-24.7) months after MBC diagnosis and 2.1 (IQR 1.0-8.1) months after brain metastasis. The mean (SD) number of metastatic sites, including brain and other extracranial sites, at the index was 3.5 (1.9). Apart from brain metastasis, most patients had metastasis to the bone (64.6%), distant lymph node (40.2%), lung (39.0%), and liver (35.4%).

**Table 2.** Clinical characteristics at baseline.^a^

Characteristic	Total (*N* = 82)	ABE + AI (*n* = 28)	ABE + FUL (*n* = 40)
Stage at initial breast cancer diagnosis, *n* (%)			
Stage 1-2	37 (45.1)	9 (32.1)	23 (57.5)
Stage 3	25 (30.5)	6 (21.4)	11 (27.5)
Stage 4	15 (18.3)	11 (39.3)	<5
Not documented	5 (6.1)	<5	<5
ECOG PS, *n* (%)^b^			
0	24 (29.3)	9 (32.1)	14 (35.0)
1	31 (37.8)	8 (28.6)	14 (35.0)
2+	11 (13.4)	5 (17.9)	<5
Missing	16 (19.5)	6 (21.4)	9 (22.5)
Menopausal status, *n* (%)			
Postmenopausal	63 (76.8)	18 (64.3)	33 (82.5)
Pre/perimenopausal	9 (11.0)	<5	<5
Unknown/not applicable	10 (12.2)	6 (21.4)	<5
CCI, *n* (%)			
0	50 (61.0)	19 (67.9)	24 (60.0)
1	22 (26.8)	7 (25.0)	10 (25.0)
2+	10 (12.2)	<5	6 (15.0)
BMI in kg/m^2^, mean (SD)^c^	28.8 (7.7)^d^	27.7 (7.4)^d^	30.4 (8.4)^d^
BMI category, *n* (%)^c^, kg/m^2^			
Obese (≥30)	25 (30.5)	6 (21.4)	17 (42.5)
Overweight (25 to <30)	25 (30.5)	11 (39.3)	9 (22.5)
Normal weight (18.5 to <25)	23 (28.0)	7 (25.0)	11 (27.5)
Underweight (<18.5)	<5	<5	<5
Missing	7 (8.5)	<5	<5
Metastasis-related variables			
Time in months from metastatic diagnosis date to index date, median (IQR)	5.5 (1.8-24.7)	3.7 (1.6-17.6)	5.5 (1.7-27.3)
Time in months from brain metastasis date to index date, median (IQR)	2.1 (1.0-8.1)	1.9 (1.3-5.9)	2.1 (0.94-7.5)
Number of metastatic sites, mean (SD)	3.5 (1.9)	3.4 (1.8)	3.8 (1.9)
Number of metastatic sites, *n* (%)			
1-2	27 (32.9)	10 (35.7)	11 (27.5)
3-4	33 (40.2)	11 (39.3)	17 (42.5)
5+	22 (26.8)	7 (25.0)	12 (30.0)
Brain as the first site of metastasis, *n* (%)	46 (56.1)	17 (60.7)	19 (47.5)
Number of new brain-only lesions after brain metastasis and before initiating ABE			
0	68 (82.9)	24 (85.7)	32 (80.0)
1	9 (11.0)	<5	6 (15.0)
2+	5 (6.1)	<5	<5
Number of new brain-only lesions during ABE treatment^e^			
0	68 (82.9)	23 (82.1)	32 (80.0)
1	13 (15.9)	5 (17.9)	8 (20.0)
2	<5	<5	<5
Lab-related data, *n* (%)			
*BRCA*			
Negative	56 (68.3)	21 (75.0)	26 (65.0)
Positive	8 (9.8)	<5	6 (15.0)
Missing	18 (22.0)	6 (21.4)	8 (20.0)
*PIK3CA*			
Negative	37 (45.1)	13 (46.4)	19 (47.5)
Positive	15 (18.3)	8 (28.6)	<5
Missing	30 (36.6)	7 (25.0)	17 (42.5)
*PD-L1*			
0%	23 (28.0)	10 (35.7)	9 (22.5)
<1%-19%	7 (8.5)	<5	5 (12.5)
Missing	52 (63.4)	16 (57.1)	26 (65.0)
Estrogen receptor-positive	82 (100.0)	28 (100.0)	40 (100.0)
Progesterone receptor			
Negative	12 (14.6)	≤5	7 (17.5)
Positive	69 (84.1)	25 (89.3)	32 (80.0)
Unknown	<5	<5	<5

^a^Fourteen patients received either abemaciclib monotherapy (*n* = 6) or abemaciclib in combination with other (*n* = 8). Due to the small sample size and risk of patient re-identification, the data for these subgroups were not presented.

^b^Measured 60 days before or on the index date.

^c^Measured 45 days before or on the index date.

^d^The numbers of patients were: total, 75; ABE + AI, 26; and ABE + FUL, 37.

^e^During ABE refers to between index ABE start date and index ABE end date.

Abbreviations: ABE, abemaciclib, AI, aromatase inhibitors; BMI, body mass index; BRCA, breast cancer gene; CCI, Charlson’s Comorbidity Index; ECOG PS—Eastern Cooperative Oncology Group Performance Status; FUL, fulvestrant; IQR, interquartile range; *N*, total number of patients, *n*, number of patients in the subgroup; PIK3CA, phosphatidylinositol-4,5-bisphosphate 3-kinase catalytic subunit Alpha; PDL1, programmed death ligand 1.

Overall, 82.9% of patients had 0 new brain-only lesions and 17.1% of patients had ≥1 new brain-only lesions after brain metastasis but before initiating abemaciclib treatment, as well as during index abemaciclib treatment (Table 2).

Some clinical characteristics differed numerically between the subgroups although these differences were not statistically tested. Stage 4 breast cancer was initially diagnosed in 39.3% of patients on abemaciclib + AI and in only 7.5% of patients on abemaciclib + fulvestrant (Table 2). An ECOG PS score of 2+ was present in 17.9% and 7.5% of patients on abemaciclib + AI and abemaciclib + fulvestrant, respectively. The brain was documented as one of the first sites of metastasis in 60.7% of patients on abemaciclib + AI and 47.5% of patients on abemaciclib + fulvestrant.

### Treatments administered prior to and during abemaciclib

In terms of prior treatment, 22.0% and 19.5% of patients received a CDK4/6i (palbociclib or ribociclib) or chemotherapy in the LoT prior to abemaciclib initiation in the metastatic setting, respectively. The proportion of patients in the abemaciclib + AI or abemaciclib + fulvestrant subgroups who received CDK4/6i (palbociclib or ribociclib) and chemotherapy as prior therapy were 10.7% and 25.0%, and 27.5% and 15.0%, respectively (Table 3).

**Table 3.** Treatments administered prior to and during abemaciclib.^a^

Variables, *n* (%)	Total (*N* = 82)	ABE + AI (*n* = 28)	ABE + FUL (*n* = 40)
Treatment prior to ABE LoT			
CDK4/6i (palbociclib and ribociclib)			
Yes	18 (22.0)	<5	11 (27.5)
No	41 (50.0)	16 (57.1)	17 (42.5)
NA—ABE received in 1L	23 (28.0)	9 (32.1)	12 (30.0)
Chemotherapy			
Yes	16 (19.5)	7 (25.0)	6 (15.0)
No	43 (52.4)	12 (42.9)	22 (55.0)
NA—ABE received in 1L	23 (28.0)	9 (32.1)	12 (30.0)
Radiation therapy and local surgery to the brain after diagnosis and before initiating ABE			
None	16 (19.5)	<5	10 (25.0)
Yes	66 (80.5)	24 (85.7)	30 (75.0)
WBRT	21 (25.6)	5 (17.9)	12 (30.0)
SRS	18 (22.0)	8 (28.6)	9 (22.5)
Others^b^	27 (32.9)	11 (39.3)	9 (22.5)
Radiation to extracranial metastatic sites after diagnosis and before initiating ABE			
Yes	9 (11.0)	<5	5 (12.5)
No	73 (89.0)	25 (89.3)	35 (87.5)
Radiation therapy and local surgery to the brain during ABE			
None	48 (58.5)	17 (60.7)	22 (55.0)
Yes	34 (41.5)	11 (39.3)	18 (45.0)
WBRT	5 (6.1)	<5	<5
SRS	17 (20.7)	5 (17.9)	9 (22.5)
Others^b^	12 (14.6)	<5	6 (15.0)

NA refers to no preceding LoT. Preceding LoT is defined as index line number (the LoT where the ABE start date falls in) -1.

^a^Fourteen patients received either abemaciclib monotherapy (*n* = 6) or abemaciclib in combination with other (*n* = 8). Due to the small sample size and risk of patient re-identification, the data for these subgroups were not presented.

^b^Others included patients who received combinations of craniotomy/metastasectomy, SRS, WBRT, intrathecal chemotherapy, and other types of radiation therapy or surgery.

Abbreviations: 1L, first-line; ABE, abemaciclib; AI, aromatase inhibitors; FUL, fulvestrant; LoT, line of therapy; *N*, total number of patients; *n*, number of patients in the subgroup; SRS, stereotactic radiosurgery; WBRT, whole brain radiation therapy.

Overall, the majority of patients (80.5%) received treatment to the brain (radiation or surgery) after brain metastasis diagnosis and before abemaciclib initiation. In the abemaciclib + AI and abemaciclib + fulvestrant subgroups, 85.7% and 75.0% of patients, respectively, received treatment to the brain. Whole brain radiation therapy (WBRT; 25.6%) and SRS (22.0%), or a combination of treatments were the most common treatments to the brain (Table 3). Overall, the majority of patients (89.0%) did not receive radiation therapy to extracranial metastatic sites prior to abemaciclib initiation. Among the 9 patients who received radiation therapy to extracranial metastatic sites, external beam radiation therapy was the most common method (*n* = 5; 55.6%).

During the abemaciclib treatment period, 34 patients (41.5%) received concurrent radiation treatment to the brain; the most common being SRS (20.7%) and WBRT (6.1%; Table 3). Among the 34 patients, 5 (14.7%) also received extracranial radiation during abemaciclib treatment. On the other hand, 23 of the 34 patients (67.6%) with concurrent radiation treatment received intracranial radiation prior to abemaciclib.

### Abemaciclib treatment characteristics

Overall, 28.0%, 32.9%, and 39.0% of patients initiated index abemaciclib in the first, second, and third and later LoTs in the metastatic setting, respectively. The most common starting dose for index abemaciclib was 150 mg twice daily (67.1%) as recommended in the label. Overall, 51.2% did not have a dose change, while 20.7% and 6.1% received a dose decrease and increase, respectively, in the index abemaciclib regimen. Treatment characteristics for the subgroups were generally similar to the overall cohort (Table 4).

**Table 4.** Abemaciclib treatment characteristics.^a^

Variables, *n* (%)	Total (*N* = 82)	ABE + AI (*n* = 28)	ABE + FUL (*n* = 40)
Number of LoT of ABE initiation			
1	23 (28.0)	9 (32.1)	12 (30.0)
2	27 (32.9)	10 (35.7)	13 (32.5)
3+	32 (39.0)	9 (32.1)	15 (37.5)
Starting dose for index ABE			
150 mg twice daily	55 (67.1)	22 (78.6)	27 (67.5)
100 mg twice daily	14 (17.1)	<5	10 (25.0)
200 mg twice daily	6 (7.3)	<5	<5
Other doses^b^	<5	<5	<5
Unknown/not documented^c^	<5	<5	<5
Dose change between initial and subsequent ABE			
No dose change	42 (51.2)	14 (50.0)	19 (47.5)
Dose decrease	17 (20.7)	<5	12 (30.0)
Dose stayed same/held	12 (14.6)	7 (25.0)	<5
Dose increase	5 (6.1)	<5	<5
Other dose changes unknown/not documented^d^	6 (7.3)	<5	<5
Discontinuation of index ABE			
Not discontinued	17 (20.7)	9 (32.1)	5 (12.5)
Discontinued	65 (79.3)	19 (67.9)	35 (87.5)
Progression	24 (36.9)	7 (36.8)	13 (37.1)
Toxic effect of therapy	17 (26.2)	<5	9 (25.7)
Progression and toxic effect of therapy	7 (10.8)	<5	<5
Other^e^	17 (26.2)	6 (31.6)	9 (25.7)

Includes ABE episodes for index ABE only.

^a^Fourteen patients received either abemaciclib monotherapy (*n* = 6) or abemaciclib in combination with other (*n* = 8). Due to the small sample size and risk of patient re-identification, the data for these subgroups were not presented.

^b^Other doses included 150 mg once daily and 50 mg twice daily.

^c^Unknown/not documented included 150 mg not documented.

^d^Other dose changes unknown/not documented included dose changes not covered in the other categories and unknown/not documented dose changes.

^e^Other reasons for discontinuation included cancer-related symptoms not due to therapy, insufficient response, non-cancer-related medical issue, patient request, other and progression, hospice, and any other reason.

Abbreviations: ABE, abemaciclib; AI, aromatase inhibitors; FUL, fulvestrant; LoT, line of therapy; *N*, total number of patients; *n*, number of patients in the subgroup.

**Figure 1.**
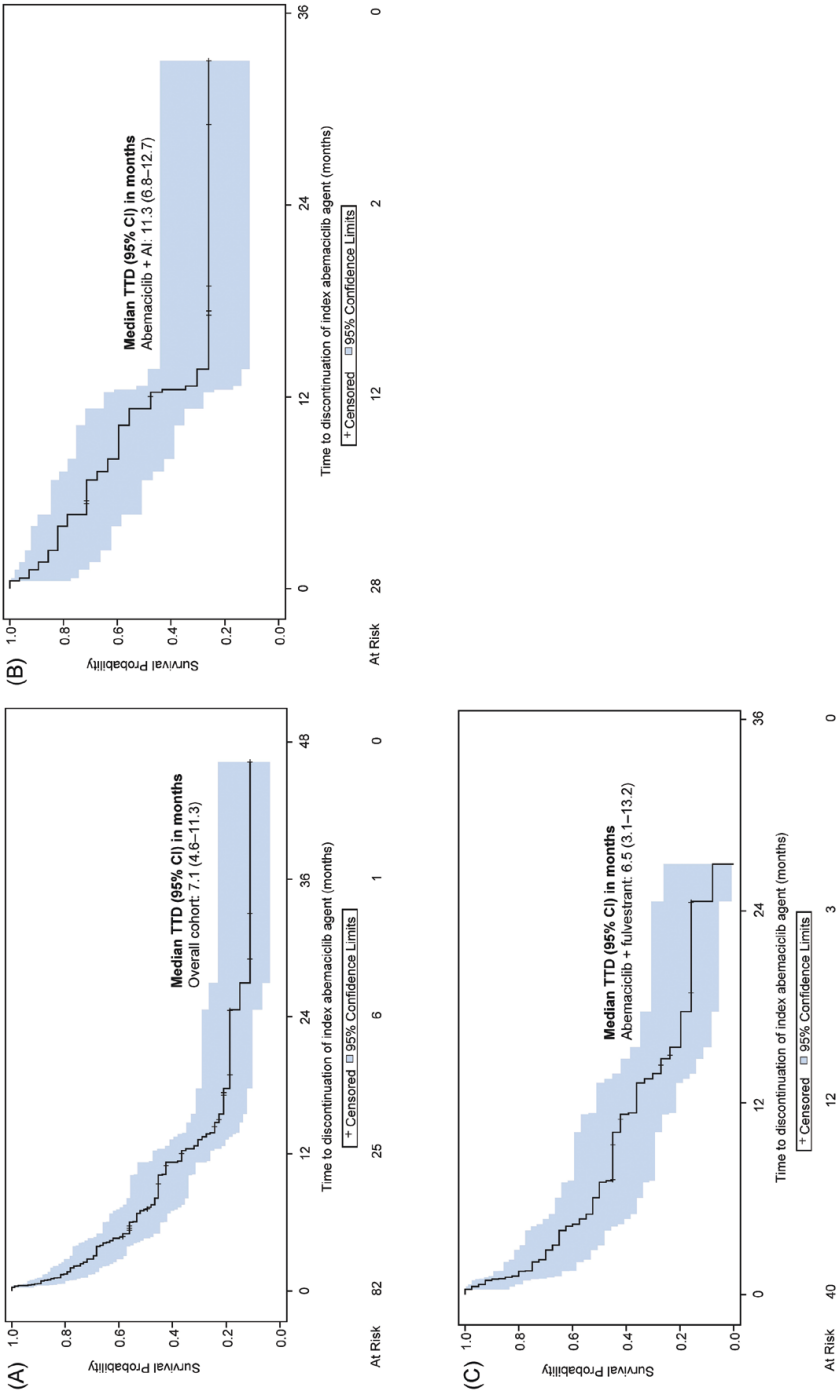
Time to treatment discontinuation in abemaciclib-treated patients. Fourteen patients received either abemaciclib monotherapy (*n* = 6) or abemaciclib in combination with other (*n* = 8). Due to the small sample size and risk of patient re-identification, the data for these subgroups were not presented. (A) The TTD for the overall population. (B, C) The TTD for the abemaciclib + AI and abemaciclib + fulvestrant subgroups, respectively. Abbreviations: AI, aromatase inhibitors; TTD, time to treatment discontinuation.

Over a median (IQR) follow-up duration of 12.8 (5.8-21.0) months, the median TTD for the overall cohort was 7.1 (95% CI 4.6-11.3) months (Figure 1A). Median TTD was 11.3 (95% CI 6.8-12.7) months in the abemaciclib + AI subgroup (Figure 1B) and 6.5 (95% CI 3.1-13.2) months in the abemaciclib + fulvestrant subgroup (Figure 1C). In the overall cohort, and abemaciclib + AI and abemaciclib + fulvestrant subgroups, 79.3%, 67.9%, and 87.5% of patients, respectively, discontinued abemaciclib treatment. The major reasons for discontinuation were disease progression (36.9%) and toxic effect of therapy (26.2%; Table 4).

### Outcomes: intracranial and extracranial

Among 82 patients, the majority received treatment to the brain before abemaciclib initiation (80.5%; Table 3). Intracranial and extracranial outcomes were evaluated in 51 and 60 of these 82 patients, respectively (Table 5). The real-world intracranial ORR was 45.1% and the real-world intracranial CBR was 62.7%; 13.7% of patients had complete response and 31.4% had partial response. The real-world extracranial ORR was 56.7% and the real-world extracranial CBR was 70.0%; 5.0% of patients had complete response and 51.7% had partial response. Supplementary Appendix 2 presents the ORR and CBR based on timing of radiation/surgical treatment to the brain.

**Table 5.** Objective response rate and clinical benefit rate among patients receiving abemaciclib with/without radiation/surgical treatment to the brain.

Variables, *n* (%)	
Intracranial assessment	Total (*n* = 51)
Complete response	7 (13.7)
Partial response	16 (31.4)
Stable disease ≥24 weeks of the index date	9 (17.6)
Stable disease <24 weeks of the index date	11 (21.6)
Radiation necrosis	<5
Progressive disease	<5
Indeterminate response	<5
Not documented	<5
Intracranial ORR	23 (45.1)
Intracranial CBR	32 (62.7)
Extracranial assessment	Total (*n* = 60)
Complete response	<5
Partial response	31 (51.7)
Stable disease ≥24 weeks of the index date	8 (13.3)
Stable disease <24 weeks of the index date	8 (13.3)
Progressive disease	9 (15.0)
Not documented	<5
Extracranial ORR	34 (56.7)
Extracranial CBR	42 (70.0)

Less than 5 patients in the intracranial and extracranial assessments did not receive any radiation/surgical treatment to the brain.

Response rate = complete response + partial response.

Clinical benefit rate = complete response + partial response + stable disease ≥24 weeks of the index date.

Abbreviations: CBR, clinical benefit rate; *n*, number of overall patients analyzed; ORR, objective response rate.

**Figure 2.**
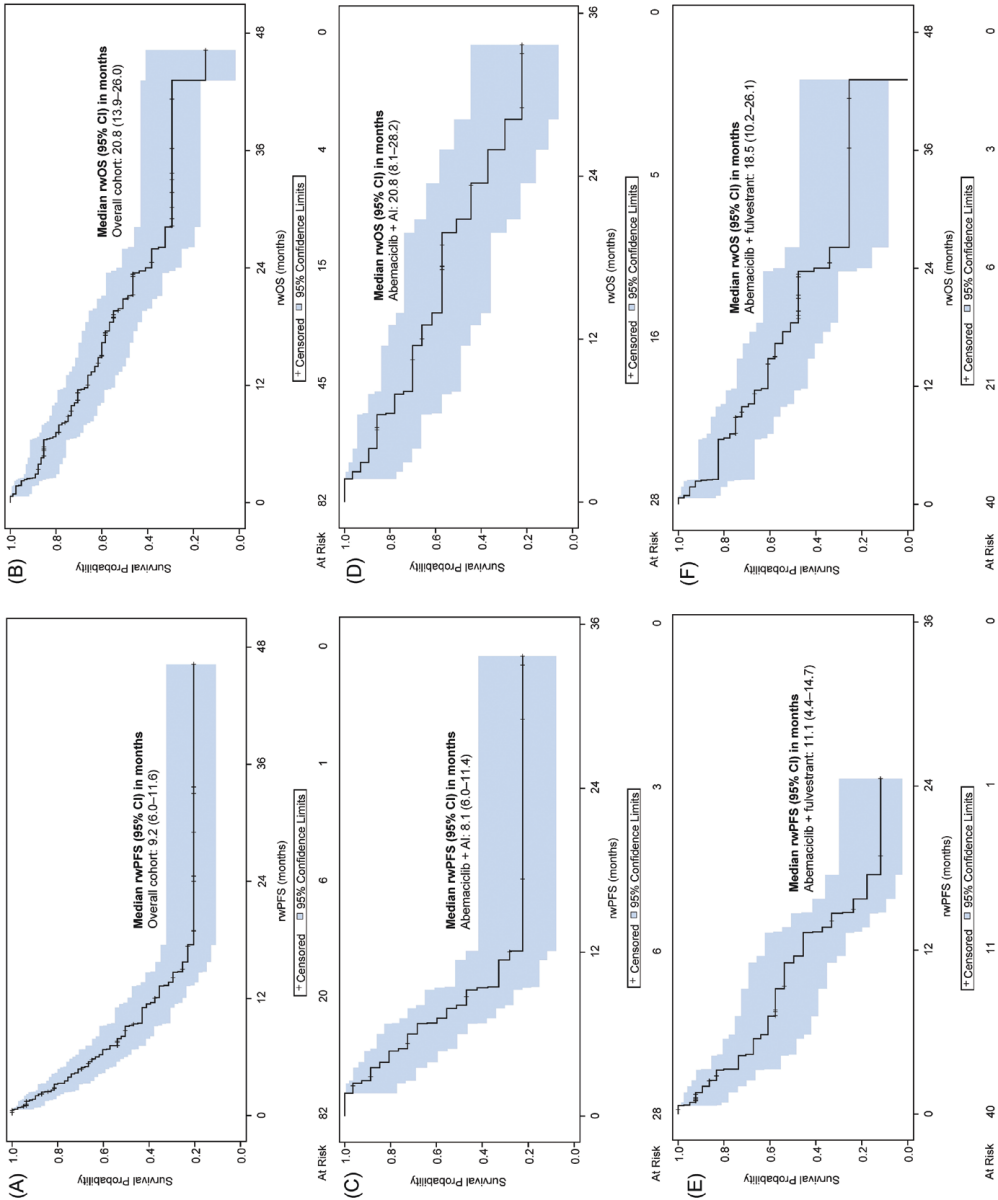
Real-world progression-free survival and real-world overall survival in abemaciclib-treated patients. Fourteen patients received either abemaciclib monotherapy (*n* = 6) or abemaciclib in combination with other (*n* = 8). Due to the small sample size and risk of patient re-identification, the data for these subgroups were not presented. (A, B) The rwPFS and rwOS for the overall population. (C, D) The rwPFS and rwOS for the abemaciclib + AI subgroup. (E, F) The rwPFS and rwOS for the abemaciclib + fulvestrant subgroup. Abbreviations: AI, aromatase inhibitors; rwOS, overall survival; rwPFS, progression-free survival.

In the overall cohort, the median rwPFS was 9.2 (95% CI 6.0-11.6) months and median rwOS was 20.8 (95% CI 13.9-26.0) months (Figure 2A and B). In the abemaciclib + AI subgroup, the median rwPFS was 8.1 (95% CI 6.0-11.4) months and median rwOS was 20.8 (95% CI 8.1-28.2) months (Figures 2C and D). In the abemaciclib + fulvestrant subgroup, the median rwPFS was 11.1 (95% CI 4.4-14.7) months and the median rwOS was 18.5 (95% CI 10.2-26.1) months (Figures 2E and F).

## Discussion

The current study aimed to describe the patient characteristics, treatment patterns, and real-world outcomes of abemaciclib-treated patients with brain metastasis secondary to HR^+^/HER2^−^ MBC. After treatment with abemaciclib, which was preceded in most cases by local control with radiation/surgery, the median TTD was 7.1 months, the intracranial ORR and CBR were 45.1% and 62.7%, and extracranial ORR and CBR were 56.7% and 70.0%, respectively. The median rwPFS and rwOS were 9.2 months and 20.8 months. Most patients in this study received treatment to the brain before abemaciclib treatment (80.5%) and abemaciclib in combination with ET (82.9%). The study was not designed to study causality, therefore, whether the benefit was due to the effect of abemaciclib, ET, and radiation/surgical treatment to the brain cannot be inferred. This retrospective study adds to the body of evidence^20^ and suggests there may be clinical benefit in patients receiving abemaciclib, ET and radiation/surgical treatment to the brain.

There are limited real-world studies focusing on outcomes in patients with brain metastasis secondary to HR^+^/HER2^−^ MBC. Minmin et al^32^ conducted a retrospective study and included a sub-cohort of patients diagnosed with HR^+^/HER2^−^ MBC receiving a CDK4/6i after brain metastasis (*n* = 163; palbociclib, *n* = 108; abemaciclib, *n* = 51; and ribociclib, *n* = 4). Consistent with the current study, in the Minmin et al study, only a few patients (9%) did not receive radiation/surgical treatment to the brain, with WBRT (40%) and SRS alone (34%) as the most common treatments to the brain. The authors reported a median central nervous system (CNS) PFS (defined as intracranial progression from the time of brain metastasis development) of 21 months, and median rwOS of 25 months. Therefore, existing evidence reports a wide range of rwOS for patients with brain metastasis and HR^+^/HER2^−^ MBC, from 9.4 months among elderly patients^4^ to 25 months as reported by Minmin et al^32^ among patients receiving CDK4/6i; the current study reported a rwOS of 20.8 months.

Abemaciclib has been evaluated previously in a phase II trial that included a cohort of patients with brain metastasis in HR^+^/HER2^−^ MBC (*n* = 58).^20^ In the trial, the intracranial and extracranial ORRs were 5.2% and 3.4%, and the intracranial and extracranial CBRs were 24.1% and 20.7%, respectively. The overall median PFS and OS were 4.4 months and 12.5 months, respectively. Investigators in the study by Tolaney et al^20^ evaluated responses uniformly as per the Response Assessment in Neuro-Oncology Brain Metastases (RANO) criteria,^33^ a standardized assessment for tumor response and progression in clinical trials. In this current retrospective study, responses were evaluated using the clinician’s documentation in a nonuniform way. Furthermore, in the published phase II trial,^20^ radiation/surgical treatment to the brain was not part of the intervention, which is likely a key contributor to the outcomes reported in this real-world study. In summary, the current real-world study is different from the phase II trial in study design, eligibility criteria, prior treatment therapy, measurement of disease progression, and use of local treatment to the brain during abemaciclib. Therefore, the results should be interpreted with caution and comparisons cannot be drawn.

Previous studies have evaluated the efficacy of ribociclib^34^ and palbociclib^35,36^ in patients with brain metastases. In a subgroup analysis of the phase IIIb CompLEEment-1 trial, the ORR and CBR were 42.9% and 62.9%, respectively, in 51 patients with CNS metastases in HR^+^/HER2^−^ MBC and treated with ribociclib + letrozole.^34^ A real-world study from China in 6 patients with brain metastases in HR^+^/HER2^−^ MBC and treated with palbociclib + ET reported an intracranial disease control rate of 50%.^35^ In a phase II basket trial, Brastianos et al^36^ evaluated the intracranial benefit of palbociclib in 15 patients with any metastatic solid tumor and CNS metastasis. Eight patients had intracranial benefit at 8 weeks after palbociclib initiation; the RANO intracranial benefit rate was 53.3%. These results suggest meaningful clinical benefits with ribociclib and palbociclib treatment for patients with brain metastases. For potential clinical utility, expanding the current analysis to include other CDK4/6i will be needed to provide guidance to clinicians to select an appropriate CDK4/6i for patients with brain metastases. Such an analysis can be conducted in the future but is beyond the scope of the current study.

The current study did not evaluate outcomes of patients who did not have brain metastases prior to abemaciclib treatment but developed brain metastases after initiating abemaciclib treatment. However, Minmin et al^32^ reported shorter median CNS PFS and rwOS in patients who received CDK4/6i both before and after brain metastasis compared to those who received CDK4/6i only after brain metastasis. Although this may suggest that CDK4/6i exposure before brain metastasis development may be associated with the development of resistance mechanisms that hamper CDK4/6i efficacy when used after brain metastasis,^32^ further research is required to explore this hypothesis.

## Limitations

The limitations of this study must be considered. This single-arm study only included patients initiating abemaciclib after brain metastases. Hence, no comparisons were made with other CDK4/6i or with patients not initiating abemaciclib. This study was also not designed to determine if the clinical benefit was due to abemaciclib, local therapy, ET, or the combination, therefore, causality cannot be inferred. Due to the small sample size of this study, its extent of analyses and potential generalizability are limited. Also, due to its retrospective nature, the EHR-derived data in this study has missing data. For example, ECOG PS data for 20% of the patients were missing. Information on active brain metastases, which would have helped to characterize the patients and contextualize the intracranial response, were also unavailable in the database. Tumor response was evaluated based on the clinician’s assessment recorded in patient charts or radiology reports, and not using criteria such as the Response Evaluation Criteria in Solid Tumors^37^ or RANO.^33^ Unfortunately, some patients’ records did not contain clinical documentation of tumor response. Although responses were analyzed based on the presence or absence of intracranial treatment before and during abemaciclib, the sample sizes are too small to draw any inferences. Furthermore, concordance of intra- and extracranial ORR and CBR was not conducted due to the small sample size, and only a proportion of patients had clinician assessment of real-world response in their charts. Lastly, detailed toxicity data were also unavailable.

## Conclusion

In this real-world study evaluating outcomes in patients receiving abemaciclib, most patients with brain metastases in HR^+^/HER2^−^ MBC received radiation/surgical treatment to the brain before abemaciclib initiation. Abemaciclib, ET, and radiation/surgical treatment to the brain demonstrated encouraging clinical outcomes in this patient population. However, the study was not designed to assess causality, so it is unclear if the benefit was due to local therapy, ET, abemaciclib, or the combination. Additional prospective clinical trial data are needed to establish the benefit of this treatment approach in patients with brain metastasis secondary to HR^+^/HER2^−^ MBC.

## Supplementary material

Supplementary material is available at *The Oncologist* online.

oyae274_suppl_Supplementary_Material

## Data Availability

The data that support the findings of this study have been originated by Flatiron Health, Inc. These de-identified data may be made available upon request and are subject to a license agreement with Flatiron Health. Interested researchers should contact < DataAccess@flatiron.com > to determine licensing terms and get the training, data dictionary, validation, and data sets. The Flatiron Health Analytic Database can be contacted at https://flatiron.com/contact/.
