# Supplementary material for: Real-world outcomes in patients with brain metastases secondary to HR+/HER2− MBC treated with abemaciclib and local intracranial therapy
Source: Oncologist. 2024 Oct 17;30(8):oyae274. doi: 10.1093/oncolo/oyae274 (PMC12395139; doi:10.1093/oncolo/oyae274)
Supplement: oyae274_suppl_Supplementary_Material [file oyae274_suppl_supplementary_material.docx]

**Supplementary information**

**Appendix 1: Selection of the study population
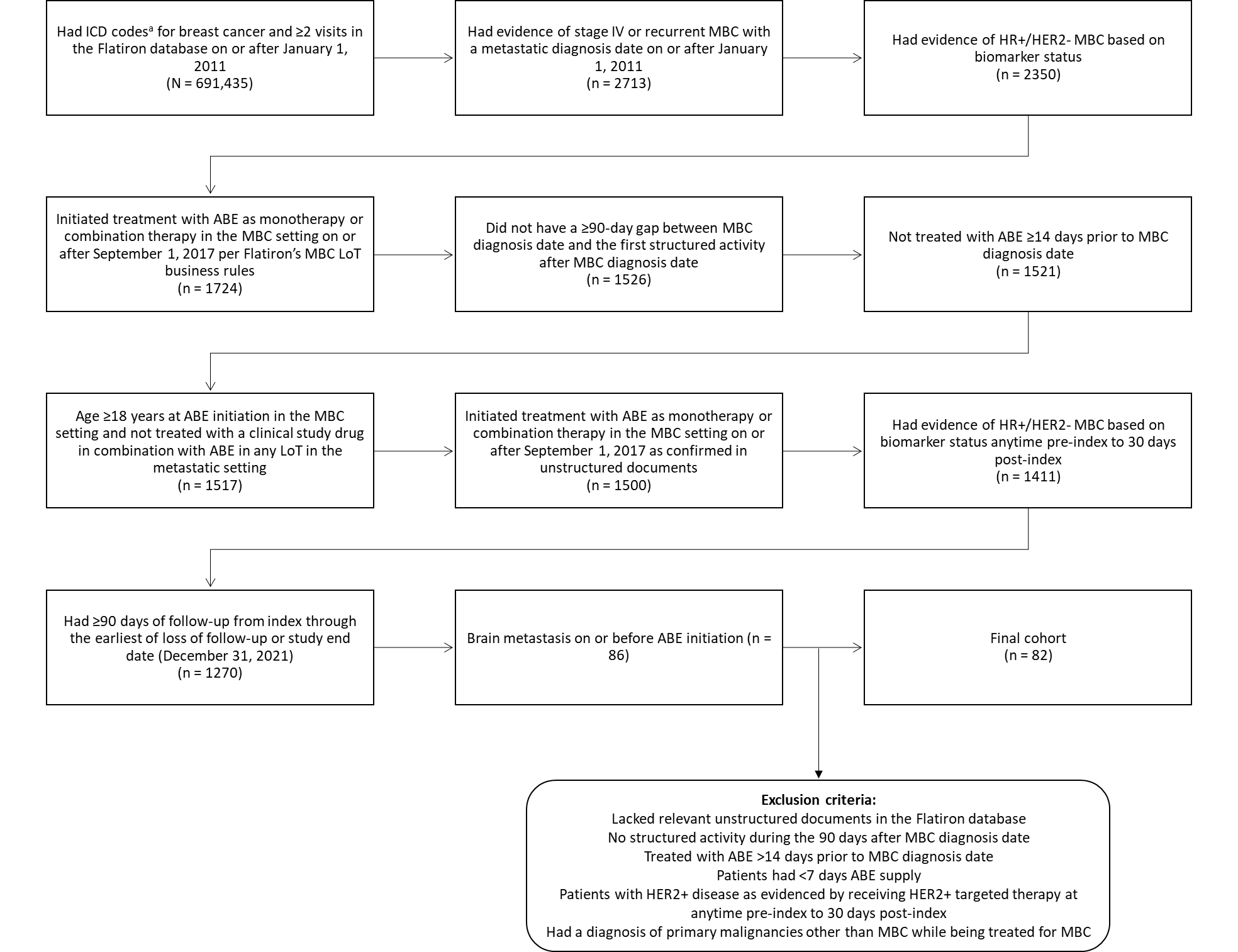
**

^a^ International Classification of Diseases, Ninth Revision 174.x or 175.x or International Classification of Diseases, Tenth Revision C50x

**Abbreviations:** ABE – Abemaciclib, HER2 - Human Epidermal Growth Factor Receptor 2, HR – Hormone Receptor, ICD – International Classification of Diseases, LoT – Line of Therapy, MBC – Metastatic Breast Cancer, N – Number of Patients in the De-identified Database Matching the Initial Inclusion Criteria, n – Number of Patients Included in the Study

**Appendix 2: Objective response rate and clinical benefit rate among patients receiving abemaciclib – breakdown based on timing of radiation/surgical treatment to the brain**

| **Variables** | **Radiation/surgical treatment before and during abemaciclib treatment** | **Radiation/surgical treatment only before abemaciclib treatment** | **Radiation/surgical treatment only during abemaciclib treatment** | **No radiation/surgical treatment before or during abemaciclib treatment** |
| --- | --- | --- | --- | --- |
| **Intracranial assessment (n)** | 18 | 22 | 7 | <5 |
| Intracranial ORR (%) | 33.3 | 50.0 | 57.1 | 50.0 |
| Intracranial CBR (%) | 50.0 | 68.2 | 57.1 | 100 |
| **Extracranial assessment (n)** | 20 | 28 | 9 | <5 |
| Extracranial ORR (%) | 60.0 | 60.7 | 55.6 | - |
| Extracranial CBR (%) | 75.0 | 67.9 | 66.7 | 66.7 |

The results need to be interpreted with caution due to the small sample size.

Response rate = complete response + partial response

Clinical benefit rate = complete response + partial response + stable disease ≥24 weeks of the index date

**Abbreviations:** CBR – Clinical Benefit Rate, n – Number of Patients Analyzed, ORR – Objective Response Rate
